# Supplementary figures and images for: Naturally occurring substitution in one amino acid in VHSV phosphoprotein enhances viral virulence in flounder
Source: PLoS Pathog. 2021 Jan 19;17(1):e1009213. doi: 10.1371/journal.ppat.1009213 (PMC7845975; doi:10.1371/journal.ppat.1009213)

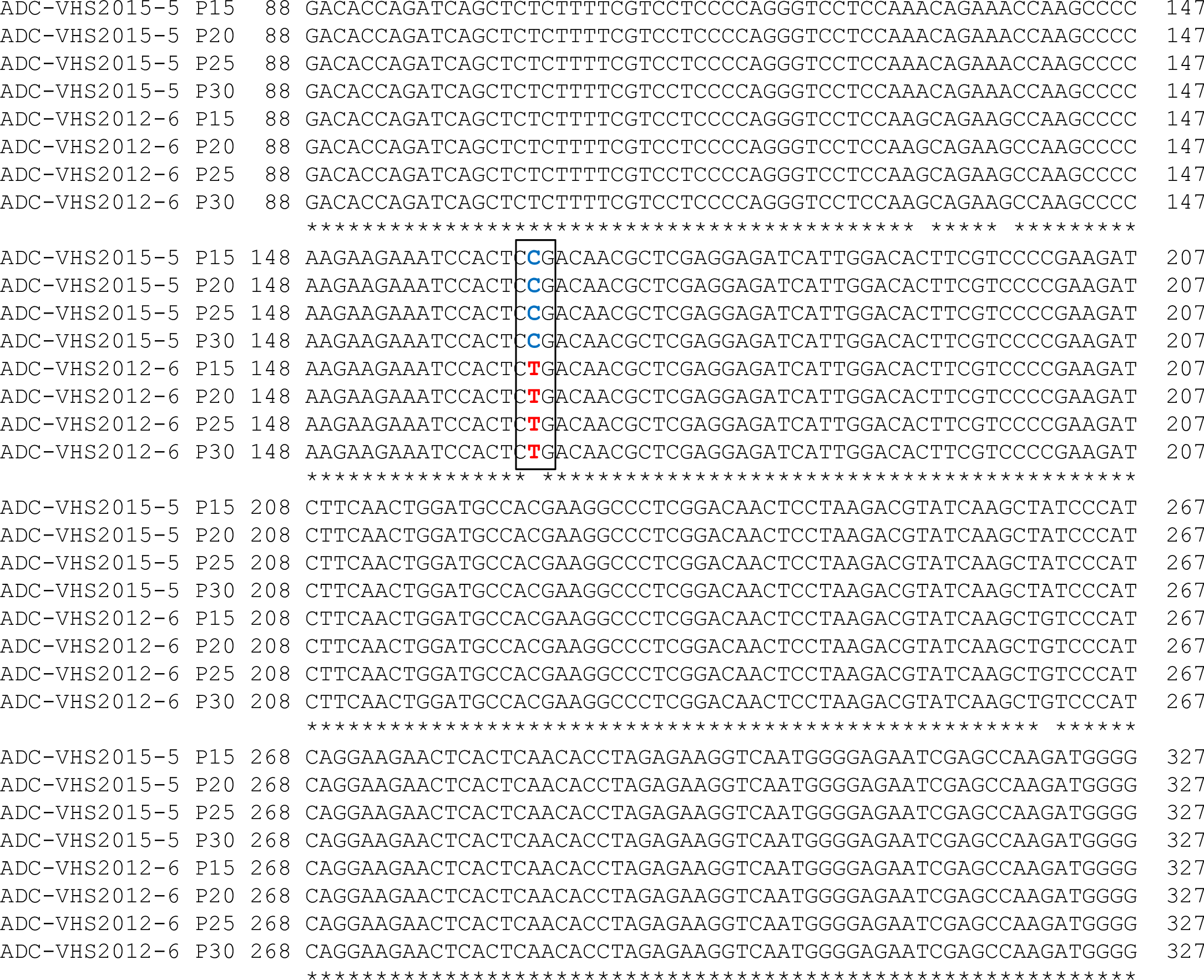

Supplement: S1 Fig — Low-virulence (ADC-VHS2015-5) and high-virulence (ADC-VHS2012-6) VHSV strains were continuously sub-cultured in HINAE cells for 30 passages. The VHSV P genes in passages 15 (P15), 20 (P20), 25 (P25), and 30 (P30) were amplified by PCR, and each base in the PCR products was sequenced an average of three or four times. The three bases in the boxed region represent the codon for the 55th amino acid residue of VHSV P. The blue bases are from ADC-VHS2015-5, and the red bases are from ADC-VHS2012-6. (TIF) [file ppat.1009213.s001.tif]

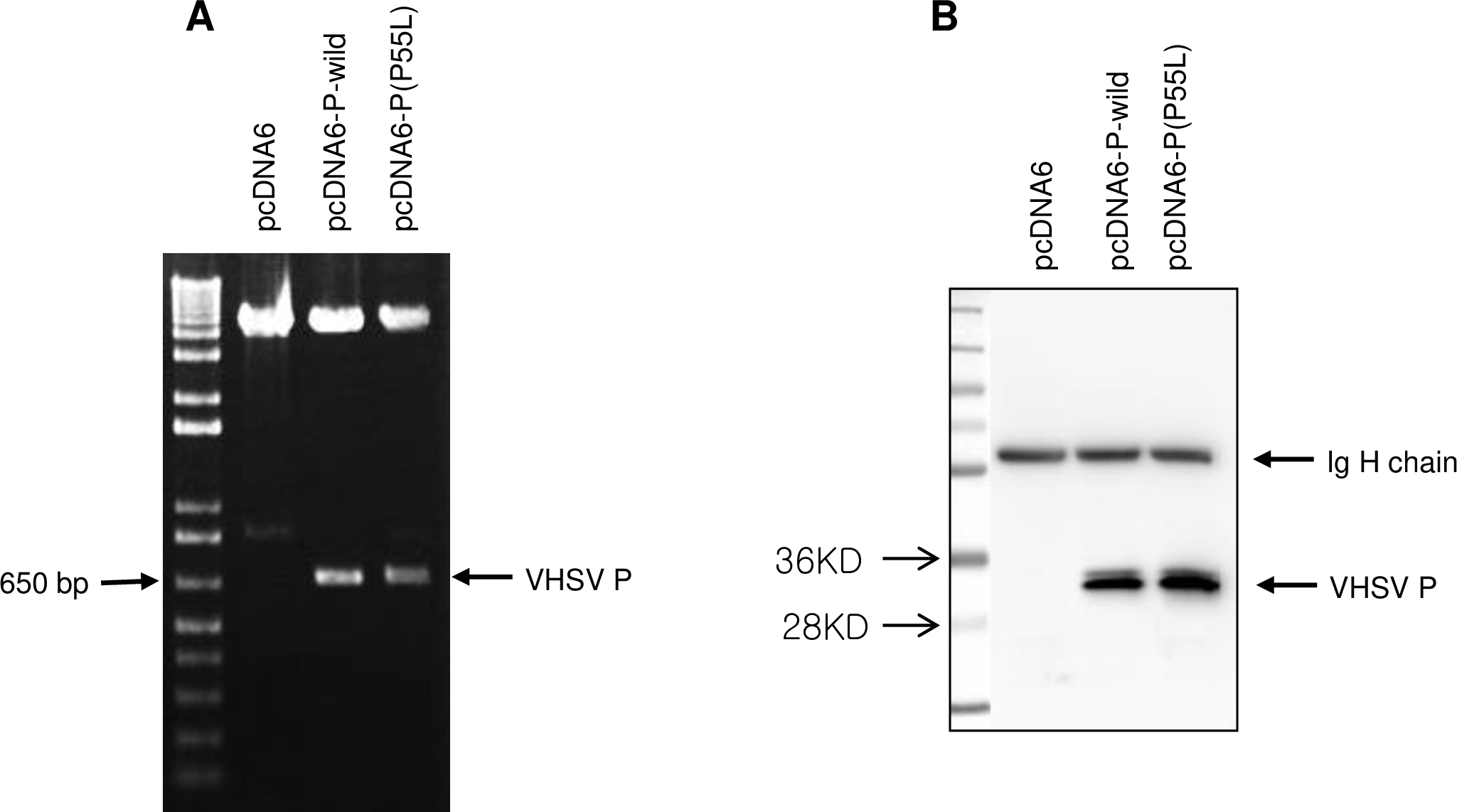

Supplement: S2 Fig — Full-length open reading frame of P genes from rVHSV-wild and rVHSV-P were PCR amplified and cloned into the HindIII/EcoRI sites of the mammalian expression vector pcDNA6/V5-His A to generate pcDNA6-P-wild and pcDNA6-P(P55L), respectively. (A) Agarose gel electrophoresis image of VHSV P gene inserts in the plasmids after cutting them with HindIII/EcoRI. (B) Each plasmid was transiently transfected into HINAE cells for 24 h, and the cell lysates were analyzed for the VHSV P protein by immunoprecipitation using an anti-V5 antibody. (TIF) [file ppat.1009213.s002.tif]

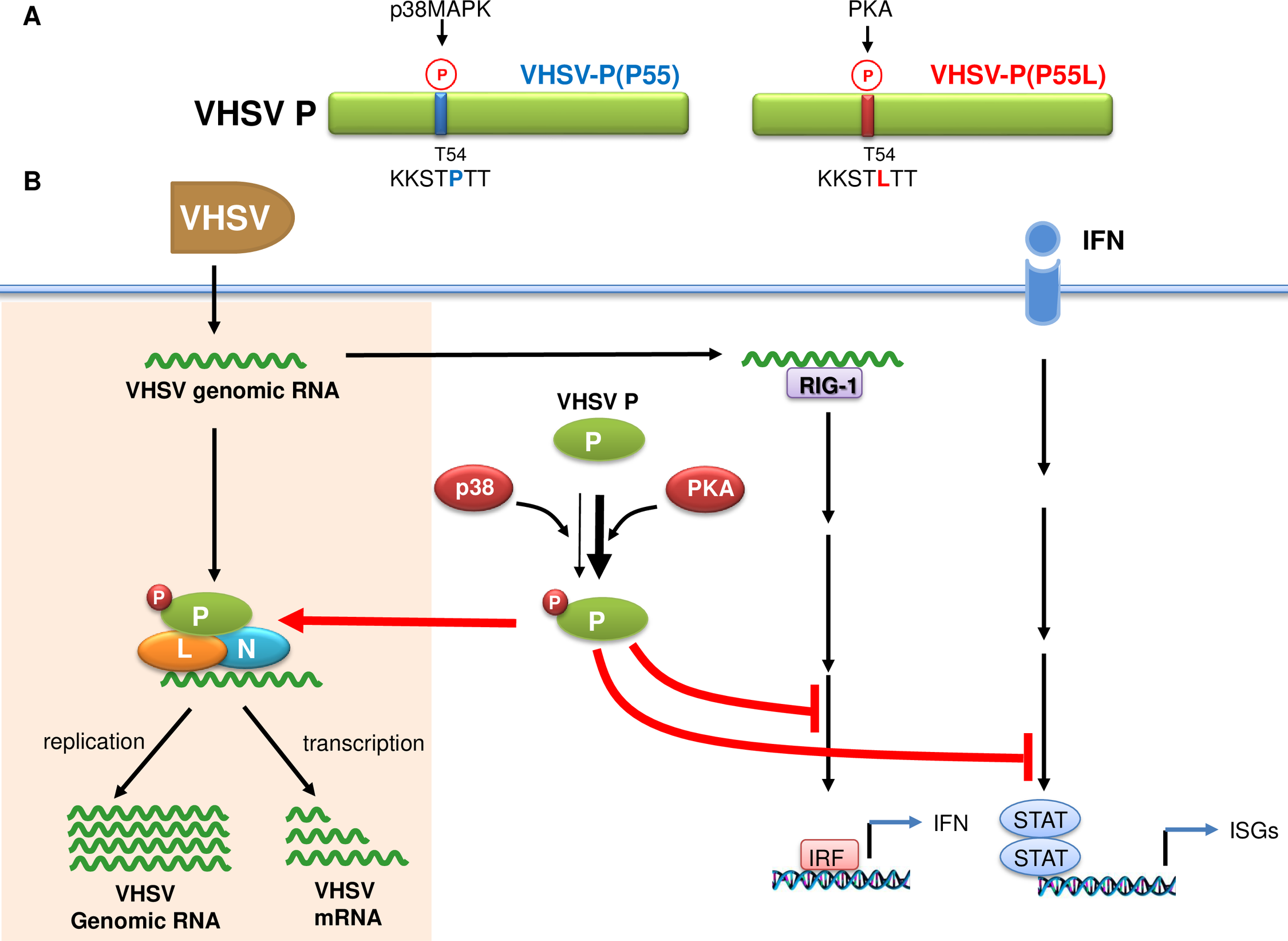

Supplement: S3 Fig — (A) Prediction of the kinase-specific phosphorylation site at T54 of the VHSV P(P55) and P(P55L) protein using the NetPhosK server. (B) Proposed model for the role of the PP55L amino acid substitution in regulating viral RNA synthesis and host IFN response. The PP55L amino acid substitution could change the kinase-specificity at T54 from p38MAPK to PKA, leading to enhanced phosphorylation of the P protein in VHSV-infected HINAE cells, which would increase the RNA polymerase activity of the VHSV L-N-P complex and block the host IFN response. (TIF) [file ppat.1009213.s003.tif]
