# Supplementary material for: Naturally occurring substitution in one amino acid in VHSV phosphoprotein enhances viral virulence in flounder
Source: PLoS Pathog. 2021 Jan 19;17(1):e1009213. doi: 10.1371/journal.ppat.1009213 (PMC7845975; doi:10.1371/journal.ppat.1009213)
Supplement: S1 Table — (DOCX) [file ppat.1009213.s004.docx]

S1. Table. VHSV strains used in this study

| VHSV strain | Genotype | Year collection | Location | Host | GenBank Accession No |
| --- | --- | --- | --- | --- | --- |
| ADC-VHS2016-2 | IVa | 2016 | Jeju, Korea | *Paralichthys olivaceus* | KY979963 |
| ADC-VHS2016-1 | IVa | 2016 | Jeju, Korea | *Paralichthys olivaceus* | KY979962 |
| ADC-VHS2015-5 | IVa | 2015 | Pohang, Korea | *Paralichthys olivaceus* | KY979961 |
| ADC-VHS2015-2 | IVa | 2015 | Jeju, Korea | *Paralichthys olivaceus* | KY979960 |
| ADC-VHS2014-2 | IVa | 2014 | Jeju, Korea | *Paralichthys olivaceus* | KY979957 |
| ADC-VHS2014-5 | IVa | 2014 | Jeju, Korea | *Paralichthys olivaceus* | KY979959 |
| ADC-VHS2014-4 | IVa | 2014 | Jeju, Korea | *Paralichthys olivaceus* | KY979958 |
| ADC-VHS2013-1 | IVa | 2013 | Pohang, Korea | *Paralichthys olivaceus* | KY979952 |
| ADC-VHS2013-9 | IVa | 2013 | Jeju, Korea | *Paralichthys olivaceus* | KY979956 |
| ADC-VHS2013-4 | IVa | 2013 | Jeju, Korea | *Paralichthys olivaceus* | KY979955 |
| ADC-VHS2013-3 | IVa | 2013 | Jeju, Korea | *Paralichthys olivaceus* | KY979954 |
| ADC-VHS2013-2 | IVa | 2013 | Jeju, Korea | *Paralichthys olivaceus* | KY979953 |
| ADC-VHS2012-11 | IVa | 2012 | Jeju, Korea | *Paralichthys olivaceus* | KY979951 |
| ADC-VHS2012-10 | IVa | 2012 | Jeju, Korea | *Paralichthys olivaceus* | KY979950 |
| ADC-VHS2012-9 | IVa | 2012 | Jeju, Korea | *Paralichthys olivaceus* | KY979949 |
| ADC-VHS2012-7 | IVa | 2012 | Jeju, Korea | *Paralichthys olivaceus* | KY979948 |
| ADC-VHS2012-6 | IVa | 2012 | Jeju, Korea | *Paralichthys olivaceus* | KY979947 |
| ADC-VHS2012-5 | IVa | 2012 | Pohang, Korea | *Paralichthys olivaceus* | KY979946 |
