# Supplementary material for: Naturally occurring substitution in one amino acid in VHSV phosphoprotein enhances viral virulence in flounder
Source: PLoS Pathog. 2021 Jan 19;17(1):e1009213. doi: 10.1371/journal.ppat.1009213 (PMC7845975; doi:10.1371/journal.ppat.1009213)
Supplement: S4 Table — (DOCX) [file ppat.1009213.s007.docx]

S4. Table. PCR primers used in this study

| Primers | |  | Sequences(5'-3') |
| --- | --- | --- | --- |
| Gene expression analysis | IFN | F | GGCCACATTCACGCAATCAC |
|  |  | R | TGCAGGTGTCTATGTGGCTA |
|  | Mx | F | GTCCATGCTGAGAACCCAGT |
|  |  | R | TCTGTCACTCAAACTGCTGCT |
|  | ISG15 | F | TCTTCCTGAGGAATGAGAAGGG |
|  |  | R | TTGAAGTCGCTCACGGTCTC |
|  | IRF10 | F | GTACCAGGGTCAGAGGGTGA |
|  |  | R | GGTCCATAGATGCGTTCGTT |
|  | IRF4A | F | AAGAAACGACCTCGACAGGA |
|  |  | R | TGAGTCTGCAGAGCTGGGTA |
|  | IRF1A | F | CCAGAGGAGCAGGAACAGAC |
|  |  | R | TCTGTTCCCCACTTTGGTTC |
|  | IRF8 | F | CCAACAAGCTCTGGTGACCT |
|  |  | R | CCTGGATGTTACAGCCTTCGT |
| P gene cloning | VHSV P | F | CCCAAGCTTATGACTGATATTGAGATGAG |
|  |  | R | GCGAATTCCTCCAACTTGTCCAACTCCG |
| Recombinat VHSV sequence confirmation | P-P55L | F | CGATGACGACTACCCAGGGGAC |
|  |  | R | TAAGTCACACTCCCATGTCT |
|  | G-T71I | F | CCAGGTCGATAAGATCTGCATG |
|  |  | R | CGGTCTTGATCCATTCTGTCC |
|  | L-Q1079R | F | CCTCATCTCAAGACGTCTCAGTTG |
|  |  | R | TGTCATTCTCCCCGAGTCCATTCT |
| Underlined sequences, *Hind*III or *EcoR*I site | | | |
