# Supplementary material for: Naturally occurring substitution in one amino acid in VHSV phosphoprotein enhances viral virulence in flounder
Source: PLoS Pathog. 2021 Jan 19;17(1):e1009213. doi: 10.1371/journal.ppat.1009213 (PMC7845975; doi:10.1371/journal.ppat.1009213)
Supplement: S5 Table — (DOCX) [file ppat.1009213.s008.docx]

S5. Table. PCR primers used to generate recombinant VHSVs

| Primers | | Sequences(5’-3’) |
| --- | --- | --- |
| P(P55L)-SDM | F | CAAGCCCCAAGAAGAAATCCACTCTGACAACGCTCGAGGAGATCATTG |
|  | R | CAATGATCTCCTCGAGCGTTGTCAGAGTGGATTTCTTCTTGGGGCTTG |
| G(T71I)-SDM | F | GTCCCCATGAGTTCGAGGACATAAACAAGGGCTTGGTCTCTGTCCCAG |
|  | R | CTGGGACAGAGACCAAGCCCTTGTTTATGTCCTCGAACTCATGGGGAC |
| L(Q1079R)-SDM | F | GAAACACTCTGGTCGTGCTCAACCCGACAGGCCAAAAAACTCAGGG |
|  | R | CCCTGAGTTTTTTGGCCTGTCGGGTTGAGCACGACCAGAGTGTTTC |
