# Supplementary material for: Naturally occurring substitution in one amino acid in VHSV phosphoprotein enhances viral virulence in flounder
Source: PLoS Pathog. 2021 Jan 19;17(1):e1009213. doi: 10.1371/journal.ppat.1009213 (PMC7845975; doi:10.1371/journal.ppat.1009213)
Supplement: Attachment — Submitted filename: Response to Reviewers.doc [file ppat.1009213.s009.doc]

Dear

Dr. Benhur Lee, Section Editor

Dr. Kasturi Haldar, Editor-in-Chief

Dr. Michael Malim, Editor-in-Chief

The comments of the reviewers were very helpful to me. So I could improve my manuscript by modification according to the comments. In addition, I prepared response sheets in answer to the comments of the referees and enclose them with this letter.

I hope everything is going well for you.

Sincerely yours

Jeong Woo Park

Department of Biological Sciences

University of Ulsan

Ulsan 680-749, Korea

E-mail: jwpark@ulsan.ac.kr

**Major Issues: Key Experiments Required for Acceptance**

**Reviewer #1:**

***Comment***: Although the work was a bit phenomenological with respect to the impact of the mutation on P function, the authors focused on the impact on IFNs and ISG expression. The data in figures 6 and 7 showed that at certain time points, the impact of the mutant virus (or protein) impacted innate immune gene induction. The authors hypothesized that the mutant P protein suppressed innate immune gene induction more effectively, thus allowing more viral replication at early stages, giving the virus a head start on growth as compared to the control virus and allowing more rapid growth and, ultimately, mortality.

While this is perfectly fine as a hypothesis, the authors spend a bit too much effort attributing this to the anti-host function of P. But the data showing a dramatic impact of this mutant on viral RNA synthesis, particularly + strand, suggests that it would potentially alter all viral proteins, any one of which could be impacting host function (multiple have been implicated in this role). In other words, the impact on viral gene transcription could be the primary impact of the mutation, with the subsequent effects indirectly mediated by the other viral proteins/genes, especially if the mutation alters the normal balance or kinetics of anti-host gene induction.

A simple but important experiment to share with the reader along these lines is a Western Blot showing the accumulation of viral proteins post-infection, beginning at early timepoints. This could be done in multiple ways in multiple cells systems that the authors have developed, and would provide supporting data one way or another to direct future studies. It is appreciated that the authors feel that rapid growth alone cannot alter host suppression, but altered anti-host gene expression kinetics could. A pan-VHSV antibody that detects all proteins would allow this experiment to be conducted rapidly, and perhaps expand the possible interpretations, or support the hypothesis proposed by the authors.

***Response***: Because of lack of suitable antibodies specific to VHSV proteins, we could not determine the effect of the P^P55L^ amino acid substitution on the expression of VHSV proteins. However, as you suggested, it is possible that the primary impact of the P^P55L^ amino acid substitution could be the enhancing the viral gene transcription, which can lead to increase in expression of viral proteins. The increased expression of viral proteins may in turn inhibit the host antiviral immune response. We described this possibility in the “Discussion” section with four additional references.

**Reviewer #2**: no further experiments are required.

**Minor Issues: Editorial and Data Presentation Modifications**

**Reviewer #1:**

***Comment***: Figure 3 - please label the viruses used in the studies the same in the graph inset

***Response***: According to your comment, we used the same label of the viruses in the graph inset of Fig. 3. We also used the same label of the viruses in the graph inset of Fig. 2C.

***Comment***: Within the text that discusses “Effects of the substitution on the expression of IFN genes in vitro and in vivo” the authors claim that at 12 and 24hpi the expression of IFN and Mx in P55L infected cells increased to levels similar to WT infected cells. This is not supported by the data in figure 6a where the 24 hpi data are not very similar to wt (although they are at 12 hpi). The narrative simply needs to align with the data better.

***Response***: According to your comment, we modified the sentence by describing the expression pattern of IFN genes at 12 h and 24 h more precisely based on the data in Fig. 6A.

**Reviewer #2:**

***Comment 1***: Results line 1 say the 18 strains were isolated from olive flounder during "an outbreak" of VHS in Korea between 2012-2016. The words "an outbreak" suggest they were all from the same location, which is not a very broad sampling of virus types. However, a look at the paper cited (#43) shows the strains were from multiple VHS outbreaks at several different locations over the 4 years. Revise wording to indicate the strains were from multiple outbreaks, because the broader sources of the virus strains tested makes the work much more significant. Also, I would say that they were all from cultured flounder (I believe none were from wild fish?).

***Response***: According to your comment, we replaced the “an outbreak” with “multiple outbreaks”. In addition, to make it sure that all VHSV strains were from cultured flounder, we added “cultured” in front of “olive flounder”.

***Comment 2***: Results section 1, lines 8-10. Here the description of defining the virulence phenotypes is not quite clear. It is important that readers understand the virulence trial was conducted 4 times and the average of the mortality observed in all trials was used to define your phenotypes. I suggest revising to "Based on the average mortality observed in four independent experiments, we selected 7 VHSV strains that caused 70-75% cumulative mortality (high virulence strains) and 6 VHSV strains that caused 35-60% cumulative mortality (low virulence strains) ....
Please check on the number of low virulence strains - that sentence in the text now says 5 low virulence strains, but in Table 1 I think there are 6. In Table 1 I would suggest adding another column on the right to indicate the virulence phenotype (high, moderate, low) - this is to help readers easily follow which strains you have defined as being in each group.

***Response***: According to your comment, we replaced the original sentence with “Based on the average mortality observed in four independent experiments, we selected 7 VHSV strains that caused 70-75% cumulative mortality (high-virulence strains) and 6 VHSV strains that caused 31.25-57.5% cumulative mortality (low-virulence strains) in olive flounder (Fig. 1 and Table 1).”. We also corrected the number of low virulence strains from 5 to 6. In addition, we added one more column on the right to indicate the virulence phenotype.

***Comment 3***: Results section 5 on Stability of PP55L in VHSV during viral replication. Here I think the notation should be changed because the serial passage study involves naturally occurring high and low virulence strains that differ at P55, not mutated strains. This is really testing variants PP55 and PL55, and we do not know which was ancestral. The notation PP55L indicates a mutation from P to L, which is appropriate for the in vitro mutagenesis of the infectious clone, where the original clone had a P and it was mutated to L. But the natural strains should be presented as variants without assuming you know the direction of mutation that created the polymorphism (either could have been ancestral). To make this clear in the discussion I would suggest revising Discussion line 7 to "...this single amino acid polymorphism was stably maintained for 30 passages of naturally occurring variant strains."

***Response***: According to your comment, we replaced the original sentence in the “Discussion” section with “this single amino acid polymorphism was stably maintained for 30 passages of naturally occurring variant strains.”.

***Comment 4***: Results section 8 on Ectopic expression of PP55L generated some really fascinating results shown in Figure 5, but it seems the written text in the results section does not describe the results in the Figure fully.

***Comment 4a***: Please explain in the methods what anti-V5 antibody is (monoclonal or polyclonal?), and say where you got it from.

***Response***: The anti-V5 antibody used in this study was monoclonal antibody purchased from GenWay Biotech Inc. According to your comment, we added this information in the “Materials and Methods” section.

***Comment 4b***: In Fig. 5B it looks like expression of PP55L increased expression of + RNA for both the low and high virulence strains, relative to the expression levels with the pcDNA6 empty vector, but the results section mentions only an increase for the low virulence strain. Was there also an increase in expression of for the high virulence strain? This might seem logical since it would be an increased dose effect of having more PP55L (from both the high virulence strain and the ectopic expression). It seems this should have been tested statistically, and if it is significant there should be a bracket to indicate that also.

***Response***: As you indicated, there was also a significant increase in expression of (+)RNA for the high virulence VHSV strain. We added this information to the “Results” section and also added the result of statistical analysis to the graph.

***Comment 4c***: Figure 5C appears to show a significant decrease of (-) sense RNA expression for the high virulence strain when the PP55 is expressed ectopically, but this is not mentioned in the text of the results. That text says there was no effect, which seems to contradict Figure 5C.

***Response***: As you indicated, ectopic expression of P^P55^ decreased (-) RNA synthesis of the high-virulence VHSV strain. We mentioned this in the text of the “Results” section.

***Comment 4d***: Also in Figure 5C, as in Figure 5B, it appears that ectopic expression of PP55L results in an increase of (-) RNA expression for the high virulence strain. If so this is very interesting and should be mentioned.

***Response***: According to your comment, we mentioned that ectopic expression of P^P555L^ increased (-) RNA synthesis of the high-virulence VHSV strain in the text of the “Results” section.

***Comment 5***: Results section 9 on IFN genes:

***Comment 5a***: In line 2 for the general phenomenon of viruses modulating immune responses the reviews cited are both generally for mammalian viruses. It would be useful to also cite the Purcell et al. review on Immunity to Fish Rhabdoviruses, 2012, since that is specific to the system being presented.

***Response***: According to your comment, we cited one more article (Purcell et al. Viruses 2012;4(1):140-166) reviewing on immunity to fish rhabdoviruses.

***Comment 5b***: In the next sentence, also in line 2, I would suggest revising "The P protein of rhabdoviruses ..." to "The P protein of rabies virus ...". This is because the references cited for the role of the P protein (#33-37) are all for rabies virus, but the P protein of the other model rhabdovirus, vesicular stomatitis virus, does not have the same function. Therefore, to avoid suggesting that all rhabdoviruses use the same mechanism to modulate IFN, simply revise wording to "The P protein of rabies virus...".

***Response***: According to your comment, we replaced the original sentence with “The P protein of rabies virus…”

***Comment 5c***: In lines 10-12 the sentence starting with "However, at 12 h and 24 h ..." does not describe Figure 6A-6C quite accurately. At 12 h the 2 strains are very similar for all 3 genes, but by 24 h there is an interesting shift to lower expression for all 3 genes in rVHSV-P infected cells, with significantly less for ISG15 and Mx (if I understand the statistical notation correctly). Revise wording to present the data at 24 h more clearly.

***Response***: According to your comment, we modified the sentence by describing the expression pattern of 3 genes at 12 h and 24 h more precisely based on the data in Fig. 6A-6C.

***Comment 5d***: second paragraph in Results section 9, line 3 says that samples were collected from i.p. infected flounder at 7 days. There is not 7 d time point shown in Figure 6?

***Response***: We corrected the error by deleting “7 days” from the sentence.

***Comment 5e***: second paragraph, lines 6-8, sentence starting "However, from 1 day after viral infection ...". This sentence is confusing and does not seem to describe the results in Fig. 6D and 6E well. It refers to expression of IFN1 and Mx, but there is no data for IFN1 in those figure panels. Also it says from day 1 the expression levels in rVHSV-P infected fish are "similar to those found in rVHSV-wild-infected fish", but the figure looks like it is still significantly less in 3 or 4 out of 6 panels.

***Response***: We corrected the sentence by replacing the “IFN1” with “ISG15”. In addition, we modified the sentence by describing the expression pattern of IFN genes at each time point more precisely based on the data in Fig. 6D and 6E.

***Comment 6***: Figure and legend suggestions:

***Comment 6-1***: Figure 1 - change Y axis to a maximum of 100% (there is no 120% mortality). Also indicate in the legend which experiment this is (I think it's experiment 2?), so readers can link the graph with table 1.

***Response***: According to your comment, we corrected the Y axis in Fig. 1. As you indicated, the graph in the Fig. 1 was generated from the experiment 2 in Table 1. We added this information in the legend of Fig. 1.

***Comment 6-2***: Figure 3 - either make the Y axes the same (both 100% maximum?), or note in the legend that the 2 panels have different Y axes.

***Response***: According to your comment, we made the Y axes in panel A and B the same.

***Comment 6-3***: Figure 4 - to help readers follow, note which virus strain is low virulence and high virulence. Note that the negative-sense RNA is in panels B and D, and positive-sense in A and C. then please clarify what is being compared to produce the statistical annotation - comparison of the 2 virus strains within each time point?

***Response***: According to your comment, we added the information about the virulence of two VHSV strains used in Fig. 4. We also noted that the negative-sense RNA is in panels B and D, and positive-sense in A and C. The statistical annotation was produce by comparing 2 viruses within each time point. To make it clear, we added lines between two groups which were compared to produce the statistical annotation.

***Comment 6-4***: Figure 6 please clarify what the significance levels mean in the figure. i.e. what is compared in each case? Fold-change over mock? Or Differences between the 2 virus strains within each time point?

***Response***: The statistical significance means difference between 2 viruses within each time point. To make it clear, we added lines between two groups which were compared to produce the statistical annotation.

***Comment 7***. Discussion details:

***Comment 7a***: paragraph 2, line 7 says rVHSV-P blocked induction of IFN1, ISG15, and Mx "more effectively" than rVHSV-wild. Do you have evidence to show that rVHSV-wild also blocks induction? If not, better wording might be that rVHSV-P blocks induction "relative to" rVHSV-wild.

***Response***: According to your comment, we modified the sentence by replacing “rVHSV-P blocked induction of IFN1, ISG15, and Mx more effectively than rVHSV-wild” with “rVHSV-P blocks induction of IFN1, ISG15, and Mx relative to rVHSV-wild”.

***Comment 7b***: paragraph 3, line 18. I think perhaps you mean "no inhibitors" specific to flounderp38MAPK and PKA?

***Response***: According to your comment, we added “no” to that sentence.

***Comment 7c***: paragraph 4, line 6. Since you are referring here to naturally occurring variation among strains, again I would suggest changing the notation from Pp55L to just PL55, for the reason described above in point 3.

***Response***: According to your comment, we replaced “P^P55L^“ with ” P^L55^“.

***Comment 8***: Methods section for Pathogenicity Experiments in Flounder. This seems to describe one experiment, but the results section presents 4 experiments. The fish weight is important for each experiment, and it could not have all been exactly 31.85g. How were the 4 experiments done? Did they all use the same lot of fish, or were they different lots of fish? Different sizes? same challenge does in all? Also please add that the challenge period was 14 days.

***Response***: We used different lots of fish to determine the pathogenicity of 18 VHSV isolates but used the same dose of VHSV for injection. However, we used the same lot of fish to determine the pathogenicity of 2 recombinant VHSVs. According to your comment, we added this information to the “Materials and Methods” section. In addition, we added that the challenge period was 14 days.

***Response***. We made changes in the manuscript as in the following table according to the comment of the reviewer. In addition, we made several corrections.

| **Original** | **Changes** |  |
| --- | --- | --- |
| **P7, Results,**  **Virulence of VHSV strains in olive flounder:**  Eighteen VHSV strains were isolated from olive flounder during an outbreak of VHS in Korea between 2012 to 2016 (Supplementary Table 1). … olive flounder (body weight 31.85 ± 3.89 g) were intraperitoneally injected with a 1 × 10^4^ plaque-forming units (PFU) … they varied in their virulence in olive flounder (Fig. 1 and Table 1). After four independent experiments, we selected 7 VHSV strains that caused at least 70% cumulative mortality (high-virulence strains) and 5 VHSV strains that caused less than 60% cumulative mortality (low-virulence strains) in olive flounder (Fig. 1 and Table 1)…. | Eighteen VHSV strains were isolated from cultured olive flounder during multiple outbreaks of VHS in Korea between 2012 to 2016 (Supplementary Table 1). … olive flounder (body weight 31.85 ± 3.89 g) were intraperitoneally (i.p.) injected with a 1 × 10^4^ plaque-forming units (PFU) … they varied in their virulence in olive flounder (Fig. 1 and Table 1). Based on the average mortality observed in four independent experiments, we selected 7 VHSV strains that caused 70-75% cumulative mortality (high-virulence strains) and 6 VHSV strains that caused 31.25-57.5% cumulative mortality (low-virulence strains) in olive flounder (Fig. 1 and Table 1). … | According to the comment of Reviewer 2. |
| **P7, Results,**  **Identification of amino acid sequence variations associated with high VHSV virulence in flounder:**  …Among the 95 amino acid sequence variations, we focused on those common in all 7 high-virulence VHSV strains but not in the 5 low-virulence VHSV strains…. | …Among the 95 amino acid sequence variations, we focused on those common in all 7 high-virulence VHSV strains but not in the 6 low-virulence VHSV strains…. | According to the comment of Reviewer 2. |
| **P11, Results,**  **Ectopic expression of P^P55L^ enhances mRNA synthesis and the growth of low-virulence VHSV:**  …, and then the cells were infected with either the low-virulence ADC-VHS2015-5 strain or the high-virulence ADC-VHS2012-9 strain at an MOI of 1. … ectopic expression of P^P55L^ increased the (+) RNA synthesis of the low-virulence ADC-VHS2015-5 strain (Fig. 5B). On the other hand, ectopic expression of neither P^P55^ nor P^P55L^ affected (-) RNA synthesis of the low-virulence ADC-VHS2015-5 strain or the high-virulence ADC-VHS2012-6 strain in HINAE cells (Fig. 5C). Thus, the P^P55L^ amino acid substitution can enhance the viral (+) RNA synthesis of VHSV in cells. | …, and then the cells were infected with either the low-virulence ADC-VHS2015-5 strain or the high-virulence ADC-VHS2012-6 strain at an MOI of 1. … ectopic expression of P^P55L^ increased the (+) RNA synthesis of both the low-virulence ADC-VHS2015-5 strain and the high-virulence ADC-VHS2012-6 strain (Fig. 5B). Ectopic expression of neither P^P55^ nor P^P55L^ affected (-) RNA synthesis of the low-virulence ADC-VHS2015-5 strain in HINAE cells (Fig. 5C). However, (-) RNA synthesis of the high-virulence ADC-VHS2012-6 strain was decreased in HINAE cells overexpressing P^P55^ but increased in cells overexpressing P^P55L^ (Fig. 5C). Thus, the P^P55L^ amino acid substitution can enhance the viral RNA synthesis of VHSV in cells. | According to the comment of Reviewer 2. |
| **P12-13, Results,**  **Effects of the P^P55L^ amino acid substitution on the expression of IFN genes *in vitro* and *in vivo*:**  The virulence and growth of many viruses depend on their ability to modulate the innate immune responses of infected cells [48, 49]. The P protein of rhabdovirus antagonizes type I IFN-mediated antiviral responses by inhibiting the signaling pathways for IFN induction and response [33-37]. … However, at 12 h and 24 h after infection, the expression of IFN1 and Mx in rVHSV-P-infected cells increased to levels similar to those in rVHSV-wild-infected cells (Fig. 6A–6C).  We next intraperitoneally injected olive flounder (43.9 ± 7.43g) with 2.3 × 10^5^ PFU of rVHSV-wild or rVHSV-P and analyzed the expression levels of ISG15 and Mx in kidney, spleen, and liver samples collected at 0, 1 h, 6 h, 1 day, 3 days, and 7 days after viral injection. Consistent with results obtained from the virus-infected HINAE cells, until 6 h after viral infection, rVHSV-P-infected fish expressed lower levels of ISG15 (Fig. 6D) and Mx (Fig. 6E) genes in the kidney, spleen, and liver compared to rVHSV-wild-infected fish. However, from 1 day after viral infection, the expression of the IFN1 and Mx genes in rVHSV-P-infected fish increased to levels similar to those found in rVHSV-wild-infected fish (Fig. 6D and 6E). … | The virulence and growth of many viruses depend on their ability to modulate the innate immune responses of infected cells [48-50]. The P protein of rabies virus antagonizes type I IFN-mediated antiviral responses by inhibiting the signaling pathways for IFN induction and response [33-37]. … However, at 12 h after infection, the expression of IFN1, ISG15, and Mx in rVHSV-P-infected cells increased to levels similar to those in rVHSV-wild-infected cells (Fig. 6A–6C). By 24 h after infection, their expression in rVHSV-P-infected cells shifted toward lower levels than that in rVHSV-wild-infected cells (Fig. 6A–6C).  We next i.p. injected olive flounder (43.9 ± 7.43g) with 2.3 × 10^5^ PFU of rVHSV-wild or rVHSV-P and analyzed the expression levels of ISG15 and Mx in kidney, spleen, and liver samples collected at 0, 1 h, 6 h, 1 day, and 3 days after viral injection. Consistent with results obtained from the virus-infected HINAE cells, until 6 h after viral infection, the expression of ISG15 (Fig. 6D) and Mx (Fig. 6E) was down-regulated in the kidney, spleen, and liver of rVHSV-P-infected fish, whereas their expression was up-regulated in rVHSV-wild-infected fish. However, from 1 day after viral infection, the expression of the ISG15 and Mx genes in rVHSV-P-infected fish was up-regulated and, at 3 days after viral infection, their expression in rVHSV-P-infected fish increased to levels similar to those found in rVHSV-wild-infected fish (Fig. 6D and 6E). … | According to the comment of Reviewer 1 and 2. |
| **P16, Discussion, 1^st^ paragraph:**  With the emergence of virulent strains of VHSV … Despite the inherent infidelity of the RNA polymerase of an RNA virus [50], this single P^P55L^ amino acid substitution was stably maintained for 30 passages. … | With the emergence of virulent strains of VHSV … Despite the inherent infidelity of the RNA polymerase of an RNA virus [51], this single amino acid polymorphism was stably maintained for 30 passages of naturally occurring variant strains. … | According to the comment of Reviewer 2. |
| **P16-P17, Discussion, 2^nd^ paragraph:**  Type I IFNs are induced by … to suppress innate immune responses in virus-infected cells [48, 49, 51]. … At 6 h post-infection, rVHSV-P blocked the induction of IFN1, ISG15, and Mx more effectively than rVHSV-wild in both HINAE cells and flounder tissues. … Considering the antiviral function of the IFN response [52, 53], our results suggest that … in cells infected with a low-virulence VHSV strain at 6 h post-infection. However, at 24 h post-infection, the expression of IFN1, ISG15, and Mx in rVHSV-P-infected cells and fish was similar to that in rVHSV-wild-infected cells and fish. … but the IFN response induced by rapidly growing viruses does not correlate with protection [54-57]. Consistently, … | Type I IFNs are induced by … to suppress innate immune responses in virus-infected cells [48-50, 52]. … At 6 h post-infection, rVHSV-P blocks the induction of IFN1, ISG15, and Mx relative to rVHSV-wild in both HINAE cells and flounder tissues. … Considering the antiviral function of the IFN response [53, 54], our results suggest that … in cells infected with a low-virulence VHSV strain at 6 h post-infection. However, at 12 h and 3 days post-infection in rVHSV-P-infected cells and fish, respectively, the expression of ISG15 and Mx was similar to that in rVHSV-wild-infected cells and fish. … but the IFN response induced by rapidly growing viruses does not correlate with protection [55-58]. Consistently, … | According to the comment of Reviewer 1 and 2. |
| **P17-P18, Discussion, 3^rd^ paragraph:**  … Thus, the P^P55L^ amino acid substitution enhances the transcription of VHSV. Until now, the regulation mechanisms of VHSV RNA synthesis … a complex of the viral L and P proteins for transcription and a complex of the viral L, P, and N proteins for replication [58, 59]. Both viral transcription and replication are affected by phosphorylation of the viral P protein by host kinases [60, 61]. By using NetPhosK [62], we were able to predict kinase-specific phosphorylation sites within the VHSV P protein. … Because there is no commercially available antibody recognizing a VHSV P protein phosphorylated at T54 and inhibitors specific to flounder p38MAPK and PKA, … | … Thus, the P^P55L^ amino acid substitution enhances the transcription of VHSV. It has been reported that rhabdovirus counteracts the host antiviral immune responses by using multiple viral proteins. Rabies virus N [59] and P [60] proteins suppress the host IFN response by inhibiting RIG-1 activation and blocking IRF3 phosphorylation, respectively. Rabies virus M proteins suppresses NF-kB activity and the expression of antiviral cytokines [61]. In addition, VHSV NV inhibits IFN response by reducing TBK1 phosphorylation [62]. Even though we did not analyze the effect of the P^P55L^ amino acid substitution on the expression of viral proteins, it is possible that enhanced transcription of VHSV by the P^P55L^ amino acid substitution may lead to increased expression of VHSV proteins, which plays roles in counteracting the host antiviral immune responses. Until now, the regulation mechanisms of VHSV RNA synthesis … a complex of the viral L and P proteins for transcription and a complex of the viral L, P, and N proteins for replication [63, 64]. Both viral transcription and replication are affected by phosphorylation of the viral P protein by host kinases [65, 66]. By using NetPhosK [67], we were able to predict kinase-specific phosphorylation sites within the VHSV P protein. … Because there is no commercially available antibody recognizing a VHSV P protein phosphorylated at T54 and no inhibitors specific to flounder p38MAPK and PKA, … | According to the comment of Reviewer 1 and 2. |
| **P20, Materials and Methods,**  **Viruses:**  Eighteen VHSV strains isolated from … and quantified in terms of PFU/ml using a standard plaque assay [64]. | Eighteen VHSV strains isolated from … and quantified in terms of PFU/ml using a standard plaque assay [69]. |  |
| **P20-P21, Materials and Methods,**  **Pathogenicity experiments in flounder:**  To test the pathogenicity of the VHSV strains, … were propagated and titrated on to EPC cells using a plaque assay. The fish in the tanks were intraperitoneally (i.p.) injected with a dose of 1 × 10^4^ PFU per fish or 100 μl of control medium at 11–13°C. Tanks were monitored twice daily for fish showing signs of ill health. Clinical signs of the disease and mortality were recorded daily. Virus was re-isolated from kidney and spleen homogenates … | To test the pathogenicity of the VHSV strains, … were propagated and titrated on to EPC cells using a plaque assay. Four independent experiments were conducted to determine the pathogenicity of 18 VHSV strains using different lots of fish: experiment 1, 29.61 ± 5.55 g; experiment 2, 31.85 ± 3.89 g; experiment 3 and 4, 39.7 ± 14.63 g (mean ± SD). The fish in the tanks were intraperitoneally (i.p.) injected with the same dose of 1 × 10^4^ PFU per fish or 100 μl of control medium at 11–13°C. Two independent experiments were conducted to determine the pathogenicity of 2 recombinant VHSVs using the same lot of fish (43.9 ± 7.43g). The fish were i.p. injected with different dose of recombinant viruses: experiment 1, 2.3 × 10^4^ PFU per fish; experiment 2, 2.3 × 10^5^ PFU per fish. Tanks were monitored twice daily for fish showing signs of ill health. Clinical signs of the disease and mortality were recorded daily for 14 days. Virus was re-isolated from kidney and spleen homogenates … | According to the comment of Reviewer 2. |
| **P24, Materials and Methods,**  **Cloning and expression of VHSV P protein in HINAE cells:**  Full-length cDNA of the P genes of … The expression of the P genes in HINAE cells was confirmed by a western blot analysis using an anti-V5 antibody. VHSV P gene–transfected cells were … | Full-length cDNA of the P genes of … The expression of the P genes in HINAE cells was confirmed by a western blot analysis using an anti-V5 monoclonal antibody (GenWay Biotech Inc.). VHSV P gene–transfected cells were … | According to the comment of Reviewer 2. |
| **P24-P25, Materials and Methods,**  **RNA preparation and RNA-seq, last sentence:**  Only those reads aligned against zebrafish or flounder genomes were considered in the DEG analysis with Cuffdiff [65]. | Only those reads aligned against zebrafish or flounder genomes were considered in the DEG analysis with Cuffdiff [70]. | We made a correction. |
| **P25-P26, Materials and Methods,**  **Statistical analysis:**  Differences in the expression of innate immune response genes and differences in the growth of the two VHSV strains were evaluated by one-way ANOVA. … | Differences in the expression of innate immune response genes and differences in the growth of the two VHSV strains were evaluated by Student’s t-test or one-way ANOVA. … | We made a correction. |
| **P28-35, REFERENCES**: | We added 5 references. Accordingly, list numbers of references in the text and Reference section were changed:  1-49: same as before  50: new addition  50-57🡪51-58  59-62: new addition  58-65🡪63-70 | We made a correction. |
| **P36, Figure 1 and Figure 1 legend:**  Cumulative mortality of olive flounder … and the graph represents the cumulative mortality from one of four independent experiments (n = 20 per group). | Cumulative mortality of olive flounder … and the graph was generated using the cumulative mortality from “Exp2” of four independent experiments in Table 1 (n = 20 per group).  Y axis scale in Figure:  120 🡪 100 | According to the comment of Reviewer 2. |
| **P37, Figure 2:** | The label of the viruses:  rVHSV-PGL/rVHSV-PG/rVHSV-P/rVHSV-WT 🡪 rVHSV-wild/rVHSV-P/rVHSV-PG/rVHSV-PGL | According to the comment of Reviewer 1. |
| **P38, Figure 3:** | The label of the viruses:  rVHSV-wt/rVHSV-P/Mock 🡪 rVHSV-wild/rVHSV-P/Mock  Y axis scale in Figure 3A:  60 🡪 90 | According to the comment of Reviewer 1 and 2. |
| **P39, Figure 4 and Figure 4 legend:**  … HINAE cells were infected with (A and B) ADC-VHS2015-5, ADC-VHS2012-6, (C and D) rVHSV-wild, or rVHSV-P at a multiplicity of infection of 1 PFU per cell, and cells were collected at the indicated time points. The accumulation of VHSV genome copies (negative sense) and G gene messenger RNA copies and anti-genomes (positive-sense) in the VHSV-infected cells … The results are presented as the mean ± SD of three independent experiments. *, *p*<0.05; **, *p*<0.01; ***, *p*<0.001 | … HINAE cells were infected with (A and B) low-virulence ADC-VHS2015-5, high-virulence ADC-VHS2012-6, (C and D) rVHSV-wild, or rVHSV-P at a multiplicity of infection of 1 PFU per cell, and cells were collected at the indicated time points. The accumulation of (B and D) VHSV genome copies (negative sense) and (A and C) G gene messenger RNA copies and anti-genomes (positive-sense) in the VHSV-infected cells … The results are presented as the mean ± SD of three independent experiments. *, *p*<0.05; **, *p*<0.01; ***, *p*<0.001; ns, not significant.  Statistical annotation in Figure:  Lines were added between two groups which were compared to produce the statistical annotation. | According to the comment of Reviewer 2. |
| **P40, Figure 5 and Figure 5 legend:**  The results are presented as the mean ± SD of three independent experiments. *, *p*<0.05; **, *p*<0.01; ns, not significant. | The results are presented as the mean ± SD of three independent experiments. *, *p*<0.05; **, *p*<0.01; ***, *p*<0.001; ns, not significant.  Statistical annotation in Figure:  Statistical annotation and Lines were added between pcDNA6-transfected and pcDNA6-P(P55L)-transfected cells infected by high virulence ADC-VHS2012-6 VHSV. | According to the comment of Reviewer 2. |
| **P41-P42, Figure 6 and Figure 6 legend:**  … The results are presented as the mean ± SD of three independent experiments. **, *p*<0.01; ***, *p*<0.001. not significant. (D and E) Olive flounder (43.9 ± 7.43g) were intraperitoneally injected with … cells were collected and the expression levels of (F) IFN1 and (G) Mx were determined by real-time PCR. The expression levels obtained from mock-infected cells were set to 1. The results are presented as the mean ± SD of three independent experiments. *, *p*<0.05. | … The results are presented as the mean ± SD of three independent experiments. *, *p*<0.05; **, *p*<0.01; ***, *p*<0.001; ns, not significant. (D and E) Olive flounder (43.9 ± 7.43g) were intraperitoneally injected with … cells were collected and the expression levels of (F) IFN1 and (G) Mx were determined by real-time PCR. The expression levels obtained from mock-infected cells were set to 1. The results are presented as the mean ± SD of three independent experiments. *, *p*<0.05; ns, not significant.  Statistical annotation in Figure:  Lines were added between two groups which were compared to produce the statistical annotation. | According to the comment of Reviewer 2. |
| **P46, Table 1** | One more column was added to indicate the virulence phenotype. | According to the comment of Reviewer 2. |
